# Analyse trichoskopischer Bilder mit tiefen neuronalen Netzen zur Diagnose und Aktivitätsbewertung von Alopecia areata – eine retrospektive Studie

**DOI:** 10.1111/ddg.15847_g

**Published:** 2026-01-14

**Authors:** Raffaele Dante Caposiena Caro, Victoria Orlova, Nicola Di Meo, Iris Zalaudek

**Affiliations:** ^1^ Dermatology Clinic Hospital Maggiore of Trieste University of Trieste Triest Italien

**Keywords:** Alopecia areata, Deep Learning, Dermatologie, künstliche Intelligenz, maschinelles Lernen, Trichologie, Trichoskopie, alopecia areata, artificial intelligence, Deep learning, dermatology, machine learning, trichology, trichoscopy

## Abstract

**Hintergrund und Ziele:**

Alopecia areata (AA) ist eine Autoimmunerkrankung, die Haarausfall hervorruft. Die Diagnose wird klinisch gestellt und durch Trichoskopie unterstützt. Die Trichoskopie erfordert jedoch eine Spezialausbildung. *Deep‐Learning*‐Modelle können die Diagnose und Behandlung von AA möglicherweise unterstützen. Ziel dieser Studie war die Entwicklung eines *Deep‐Learning*‐Frameworks für die Diagnose von AA und zur Bestimmung des Aktivitätsniveaus der AA.

**Patienten und Methoden:**

Anhand einer retrospektiven Analyse trichoskopischer Bilder von Patienten mit Erkrankungen der Kopfhaut und gesunden Kontrollen wurde ein zweistufiges *Deep‐Learning‐*Modell entwickelt. Stufe‐1 des Modells hatte das Ziel, eine AA‐Erkrankung von anderen Erkrankungen der Kopfhaut sowie von gesunden Kontrollteilnehmern zu unterscheiden. In Stufe‐2 sollte ein Modell für die Erkennung des Aktivitätsniveaus der AA trainiert werden, den AA‐Datensatz in aktive AA, inaktive AA und AA mit Nachwachsen der Haare einzuteilen.

**Ergebnisse:**

In Stufe‐1 wurden eine Gesamtgenauigkeit von 88,92% und ein F1‐Score von 88,17% mit einer Fähigkeit zur Unterscheidung von AA von 90,98% erzielt. In Stufe‐2 wurden eine Genauigkeit von 83,33% und ein F1‐Score von 83,36% erreicht.

**Schlussfolgerungen:**

Unsere Studie zeigt erstmals die mögliche Verwendung künstlicher Intelligenz bei der Diagnose und Stadieneinteilung der AA und ermöglicht daher genauere Diagnosen und eine bessere Versorgung.

## EINLEITUNG

Alopecia areata (AA) ist eine Haarausfall verursachende Autoimmunerkrankung ohne Narbenbildung, die durch plötzlichen, fleckförmigen und in der Regel asymptomatischen Haarausfall gekennzeichnet ist. Sie betrifft vor allem die Haarfollikel der Kopfhaut, kann sich jedoch auch auf die Körperbehaarung auswirken.^1^, ^2^ AA betrifft etwa 2% der Gesamtbevölkerung.^1^, ^2^ Allerdings wurden mehrere Subtypen der AA beschrieben, unter anderem umschriebene Alopezie, Alopecia totalis (AT), Alopecia universalis (AU), Ophiasis, Sisaipho, diffuse alopezie areata (DAA), Alopecia areata incognita (AAI) und Marie‐Antoinette‐ und Thomas‐More‐Syndrome.^1^, ^2^ Der Verlauf der AA ist variabel. Erste kahle Flecken können expandieren, zahlreicher werden oder innerhalb einiger Monate spontan unter Nachwachsen der Haare verschwinden. Bei 34–50% der Fälle findet innerhalb eines Jahres eine spontane Remission statt, obwohl es bei vielen Patienten zu Rezidiven kommt. Die Rezidivrate reicht von 30% bis 52%, wobei die meisten Rezidive (79%) innerhalb der ersten 4 Jahre auftreten.^1^, ^2^ Bei etwa 14–25% der Patienten schreitet die Erkrankung zu AT oder AU fort und eine vollständige Wiederherstellung ist selten (< 10%).^1^, ^2^

Bei AA kommt es häufig zu Nagelveränderungen. Dies gilt insbesondere für die schwereren Formen wie AU und AT. Diese Veränderungen sind oft geringfügig und asymptomatisch. Am häufigsten sind Tüpfelnägel und Trachyonychie, die jedoch oft übersehen werden. Die Prävalenz von Nagelanomalien bei Patienten mit AA wird wahrscheinlich unterschätzt. Die berichteten Raten reichen von 7% bis 66% bei einem geschätzten Mittelwert von etwa 30%.^3^ Ein weiterer signifikanter Aspekt der AA sind die psychologischen Auswirkungen, die zu einem erhöhten Risiko für Stress, Angstzuständen und Depressionen führen. Insbesondere wurde gezeigt, dass AA die Wahrscheinlichkeit, eine schwere depressive Störung zu entwickeln, um 34% steigert. Darüber hinaus sind Suizidversuche bei Patienten mit AU und DAA am häufigsten.^4^ So berichtete eine Studie von Suizidgedanken bei 60% der AU‐Patienten, mehr als dreimal höher als bei Patienten mit umschriebener Alopezie (18%).^4^, ^5^

Die Diagnose der AA erfolgt im Wesentlichen klinisch. Die Differentialdiagnosen umfassen Alopezien mit und ohne Narbenbildung sowie genetische Erkrankungen, die mit Haarausfall einhergehen.^1^, ^2^ Umschriebene AA sollte von mehreren Erkrankungen wie Tinea capitis, Trichotillomanie, temporaler triangulärer Alopezie und vernarbenden Alopezien wie Lichen planopilaris (LPP) abgegrenzt werden. Bei Fällen mit Beteiligung des Stirnhaaransatzes oder Ophiasis, muss eine frontale fibrosierende Alopezie (FFA) – ein Subtyp von LPP – sorgfältig erwogen werden. Die FFA zeigt sich als bandförmige frontotemporale Alopezie mit Verlust der Augenbrauen und, in seltenen Fällen, Beteiligung der Schläfen. Außerdem können DAA und AAI als telogenes Effluvium (TE) oder Haarausfall nach weiblichem Muster (FPHL) fehldiagnostiziert werden. Die Geschwindigkeit des Haarausfalls ist ebenfalls ein wesentlicher diagnostischer Faktor. Ein plötzliches Auftreten lässt auf TE, AA oder anagenes Effluvium (zum Beispiel induziert durch Chemotherapie) schließen, während ein allmähliches Auftreten eher für eine Alopecia androgenetica (AGA) oder eine vernarbende Alopezie (zum Beispiel LPP) typisch ist.^1^, ^2^ Aufgrund der zahlreichen Differentialdiagnosen bleibt die histologische Untersuchung der Goldstandard bei unklaren Fällen, da sie die Unterscheidung zwischen AA und anderen Alopezien ermöglicht.^1^, ^2^ Außerdem spielt die Trichoskopie, ein nichtinvasives dermatoskopisches Verfahren für die Analyse der Kopfhaut und der Haare eine entscheidende Rolle bei der Diagnose von AA.^6^ Sie unterstützt die Beurteilung der entzündlichen Aktivität, der Marker für den Schweregrad, des Ansprechens auf die Behandlung und der prognostischen Faktoren und ist daher ein essentielles Hilfsmittel in der Trichologie.^7^ Allerdings erfordert die Trichoskopie eine Spezialausbildung.^8^ Die Entwicklung Computer‐gestützter diagnostischer Verfahren könnte Dermatologen bei der Diagnose von AA, ihrer Abgrenzung von anderen Alopezien und der Identifizierung trichoskopischer Marker für das Nachwachsen der Haare und die Erkrankungsaktivität unterstützen und so zur Optimierung der Behandlung beitragen.^9^ Leichte AA mit *Severity of Alopecia Tool* (SALT) ≤ 20 wird in der Regel mit Kortikosteroiden (topisch oder intraläsional) und in seltenen Fällen mit Kontaktimmuntherapie (zum Beispiel Quadratsäuredibutylester) behandelt. Bei mittelschwerer bis schwerer AA (SALT > 20) sind systemische Therapien wie orale Kortikosteroide oder Januskinase (JAK)‐Inhibitoren indiziert.^10^, ^11^

Maschinelles Lernen (ML) und *Deep Learning* (DL) sind zentrale Komponenten der künstlichen Intelligenz (KI) und werden zunehmend in der Dermatologie eingesetzt.^5^ KI unterstützt die Diagnosevorhersage und die Beurteilung des Behandlungserfolgs.^9^ In letzter Zeit sind KI‐basierte Modelle für die Diagnose und Beurteilung des Schweregrads von AA ins Zentrum der Aufmerksamkeit gerückt. In den meisten Studien lag der Schwerpunkt auf der Klassifizierung klinischer Bilder von Kopfhaut und Haaren. Dabei zeigten sich vielversprechende Ergebnisse hinsichtlich der Unterscheidung der AA von gesunden Zuständen sowie bei der Bewertung des Schweregrads. Allerdings wurden nur in wenigen Studien KI‐Anwendungen an trichoskopischen Bildern untersucht. In einigen wurden Frameworks zur automatischen Berechnung des SALT‐Index mit Segmentierungstechniken entwickelt. In anderen lag der Schwerpunkt auf der Ermittlung von Haardichte und ‐durchmesser sowie zusätzlichen Parametern, die für die frühzeitige Diagnose von AA und anderen Haarerkrankungen von Bedeutung sind.^12^, ^13^, ^14^, ^15^, ^16^, ^17^, ^18^, ^19^, ^20^, ^21^, ^22^, ^23^, ^24^, ^25^, ^26^, ^27^, ^28^, ^29^, ^30^, ^31^, ^32^, ^33^

Ziel dieser Studie war die Entwicklung eines DL‐Frameworks anhand videodermatoskopischer Bilder zur Diagnose von AA und zur Beurteilung der Erkrankungsaktivität, um die klinische Entscheidungsfindung für medizinische Fachkräfte mithilfe geprüfter DL‐Architekturen für die Bildklassifizierung zu verbessern.

## PATIENTEN UND METHODEN

### Aufnahme der Studienteilnehmer

Diese Studie wurde retrospektiv an Bildern durchgeführt, die während der klinischen Routineversorgung von allen Patienten mit Erkrankungen der Kopfhaut erfasst wurden, die an unserer Einrichtung zwischen März 2022 und März 2024 behandelt wurden. Außerdem wurden Bilder einer Gruppe von gesunden Freiwilligen (GF) erfasst. GF waren medizinische Fachkräfte aus unserer Abteilung. Teilnehmer, die die Kriterien in Tabelle 1 erfüllten, wurden in die Studie aufgenommen. Die Teilnehmer wurden in fünf Gruppen eingeteilt: *(1)* Patienten mit aktiver AA, *(2)* Patienten mit inaktiver oder chronischer AA, *(3)* Patienten mit AA in einer Phase mit Nachwachsen der Haare, *(4)* Patienten mit anderen häufigen Erkrankungen der Kopfhaut, einschließlich Folliculitis decalvans (FD), FFA, LPP und AGA, *(5)* GF. Die AA‐Klassifizierung erfolgte auf Grundlage der trichoskopischen Befunde, wie in der Literatur beschrieben (Tabelle 2).^34^, ^35^ Entsprechend wurden die anderen Erkrankungen der Kopfhaut gemäß der einschlägigen Literatur diagnostiziert.^36^, ^37^, ^38^, ^39^, ^40^, ^41^, ^42^, ^43^, ^44^

**TABELLE 1:** Aufnahmekriterien für Studienteilnehmer.

Einschlusskriterien	‐ Erwachsene (> 18 Jahre) mit trichologischen Erkrankungen ‐ Verfügbarkeit videodermatoskopischer Bilder ‐ Unterzeichnung einer Einwilligungserklärung unseres Krankenhauses, die es uns erlaubt, klinische Daten für Forschungszwecke zu nutzen, einschließlich genetischer, biometrischer und fotografischer Daten für die klinische und epidemiologische Forschung.
Ausschlusskriterien	‐ Teilnehmer < 18 Jahre ‐ Fehlende Einwilligungserklärung (nur bei gesunden Freiwilligen)

**TABELLE 2:** Datensatz‐Einschluss‐ und ‐Ausschlusskriterien für Bilder.

Aktive Alopecia areata	‐ Gelbe Punkte ‐ Ausrufezeichenhaare ‐ Gebrochene Haare ‐ Spitz zulaufende Haare ‐ Pohl‐Pinkus‐Einschnürungen ‐ *Coudability*‐Haare
Inaktive Alopecia areata	‐ Gelbe Punkte ‐ Vellushaar ‐ Leere Follikelöffnungen ‐ Weiße Punkte
Alopecia areata mit Nachwachsen der Haare	‐ Aufrecht nachwachsende Haare ‐ *Pigtail*‐Haare ‐ Vellushaare

### Datensatz

In dieser Studie verwendeten wir einen Datensatz aus trichoskopischen Bildern, die alle während der körperlichen Untersuchungen an unserer *Ambulatory of Adnexal Diseases* von demselben Untersucher (RDC) aufgenommen wurden. Videodermatoskopische Bilder wurden mit dem Medicam 1000® Videodermatoskop (FotoFinder Systems GmbH, Deutschland; Version 3.1.3.0 [x64]) erfasst und anschließend zum Training und zur Validierung des Algorithmus eingesetzt. Digitale trichoskopische Bilder wurden von allen betroffenen Bereichen der Kopfhaut erfasst. Ein Trichologie‐Experte mit 10‐jähriger Erfahrung am *Trieste University Hospital* (RDC) definierte die Klassifizierungskriterien und überprüfte und klassifizierte jedes Bild zusammen mit zwei unabhängigen Experten (IZ und NdM). Jede Unstimmigkeit wurde durch Diskussion der Experten gelöst. Die Einschlusskriterien für die Datensatzbilder sind in Tabelle 3 aufgelistet. Der Datensatz enthält Bilder, die in drei Aktivitätsniveaus der AA eingeteilt wurden: *(1)* aktiv, *(2)* inaktiv und *(3)* mit Nachwachsen der Haare. Darüber hinaus enthält der Datensatz Bilder von vier anderen Erkrankungen der Kopfhaut: FD, FFA, LPP und AGA, sowie Bilder von GF.

**TABELLE 3:** Klassifizierung der Alopecia areata gemäß Trichoskopie‐Befunden.

Einschlusskriterien	‐ Diagnose von allen drei Experten bestätigt, ‐ Diagnose in unklaren Fällen durch Histologie bestätigt (zum Beispiel, wenn kein Expertenkonsens erzielt wurde), ‐ Bilder im Fokus, ‐ Bilder ausschließlich mit 20‐facher Vergrößerung erfasst (entspricht einem Sichtfeld von 0,903 cm^2^), ‐ Unterzeichnete Einwilligungserklärung für klinische Forschungszwecke.
Ausschlusskriterien	‐ Fälle ohne Übereinstimmung aller drei Experten und ohne histologische Bestätigung, ‐ Bilder nicht im Fokus, ‐ Bilder, die nicht mit 20‐facher Vergrößerung aufgenommen wurden (zum Beispiel 40‐fach), ‐ Duplizierte Bilder, ‐ Fehlende Einwilligungserklärung.

Wir entwickelten ein zweistufiges DL‐Framework. Stufe‐1 wurde dazu trainiert, AA von anderen Erkrankungen der Kopfhaut und GF zu unterscheiden. Für diese Stufe wurde der Datensatz in fünf Bildgruppen unterteilt: *(1)* AA, *(2)* FD, *(3)* FFA und LPP, *(4)* AGA und *(5)* GF. In Stufe‐2 entwickelten wir ein Modell zur Unterscheidung zwischen verschiedenen Aktivitätszuständen der AA. Für diesen Zweck wurde der Datensatz mit den drei AA‐Subgruppen (aktiv, inaktiv, Nachwachsen) eingesetzt.

### Normalisierung und Augmentation der Daten

**ABBILDUNG 1:**
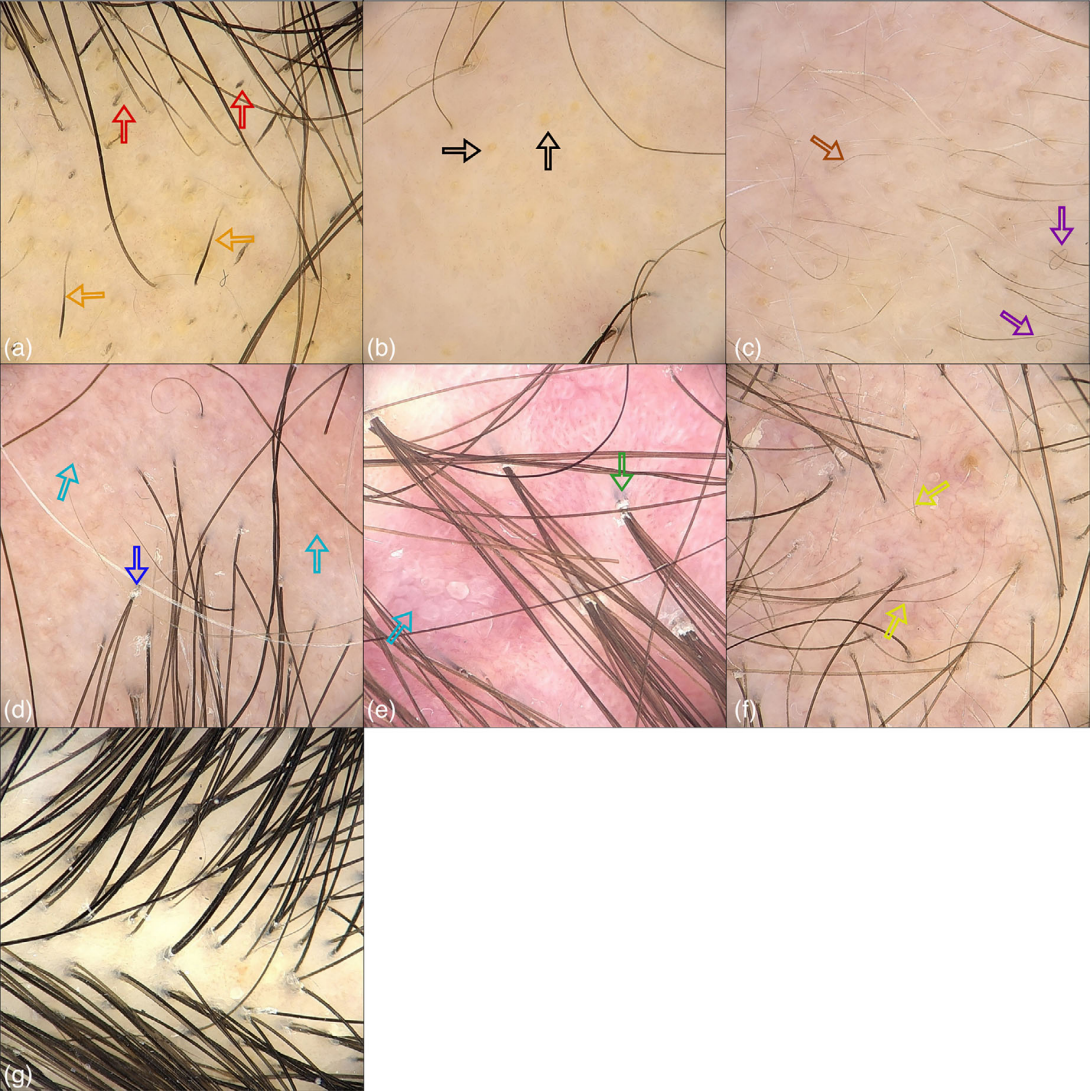
Beispielbilder von den unterschiedlichen Krankheitsklassen, die in die Algorithmen einbezogen wurden. (a) Aktive Alopecia areata, schwarze Punkte (rote Pfeile), Ausrufezeichenhaare (orangefarbene Pfeile); (b) chronisch‐inaktive Alopecia areata, gelbe Punkte (schwarze Pfeile); (c) Alopecia areata mit Nachwachsen der Haare, Vellushaare (braune Pfeile), ringförmige Haare (violette Pfeile); (d) Lichen planopilaris, perifollikuläre Schuppen (blauer Pfeil), vernarbende Bereiche (himmelblaue Pfeile); (e) Folliculitis decalvans, Büschelhaare mit perifollikulären Schuppen (grüner Pfeil), vernarbende Bereiche (himmelblaue Pfeile); (f) Alopecia androgenetica, ausdünnendes Haar (gelber Pfeil). In Stufe‐1 wurden die Bilder (a), (b) und (c) in die Klasse Alopecia areata zusammengefasst, (d) wurde als fibrosierende frontale Alopezie und Lichen planopilaris, (e) als Folliculitis decalvans, (f) als Alopecia androgenetica und (g) als gesunde Kontrolle klassifiziert. Für Stufe‐2 entsprechen die Bilder (a), (b) und (c) den drei gesonderten Klassen.

**ABBILDUNG 2:**
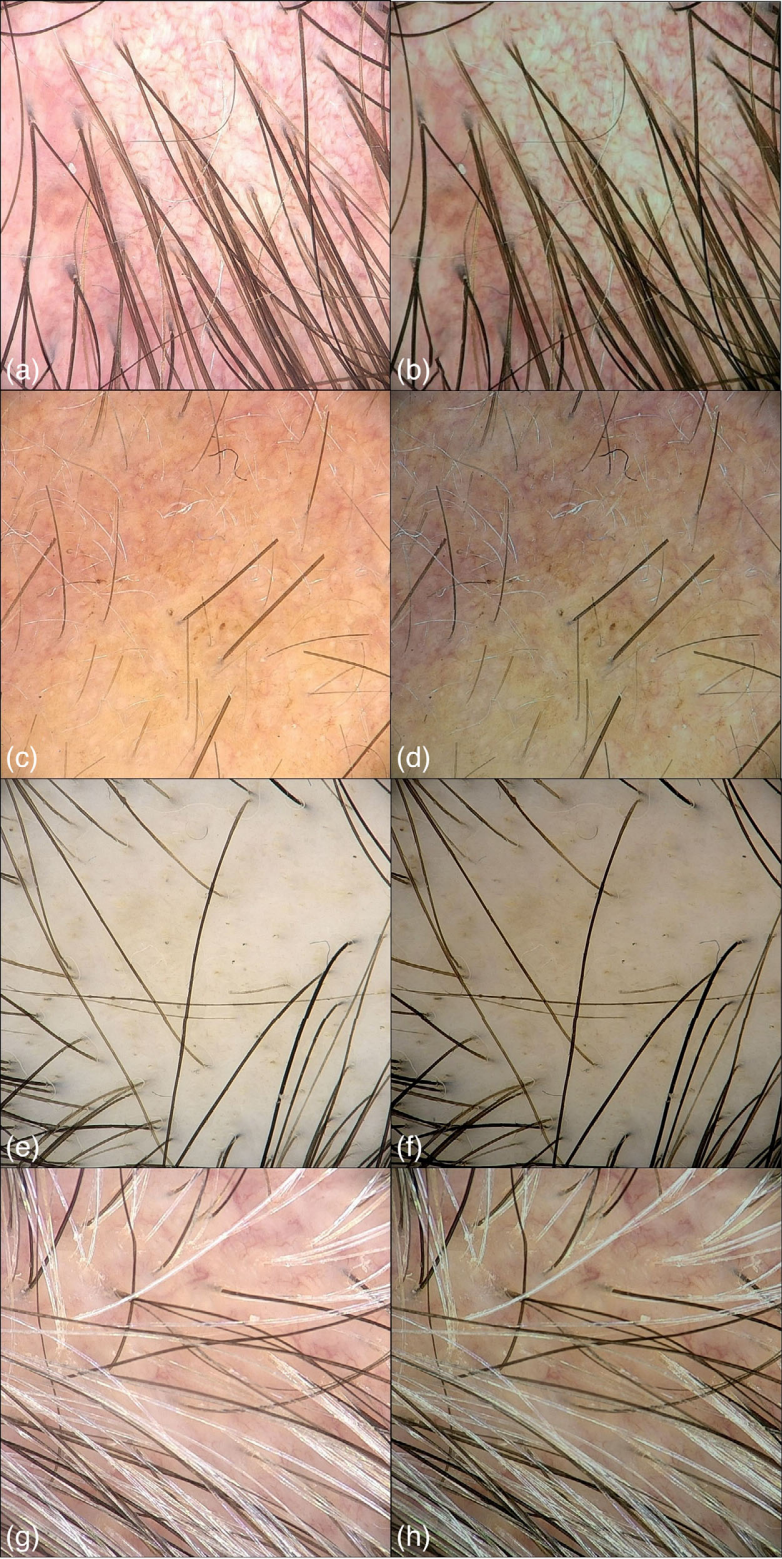
(a), (c), (e), (g) Videodermatoskopische Bilder der Kopfhaut vor der Normalisierung; (b), (d), (f), (h) videodermatoskopische Bilder der Kopfhaut nach der Normalisierung.

Es wurden nur digitale trichoskopische Bilder betroffener und gesunder Bereiche der Kopfhaut eingeschlossen, die alle Einschlusskriterien und kein Ausschlusskriterium erfüllten (Abbildung 1). Die Bilder wurden zugeschnitten, um Kameraschatten und Rauschen zu entfernen und die Klarheit und den Fokus auf die Bereiche der Kopfhaut zu verbessern. Allerdings kann die Hautfarbe aufgrund der Lichtverhältnisse und der Patientencharakteristika variieren. Um dieses Problem zu berücksichtigen, wurden die Daten normalisiert. Hauptziel der Normalisierung war es, die Variabilität hinsichtlich Helligkeit und Hautfärbung zu minimieren, um sicherzustellen, dass sich das Modell auf die relevanten Merkmale konzentriert und nicht durch Variationen bei der Beleuchtung und Hautfarbe beeinflusst wird (Abbildung 2).^25^, ^45^, ^46^ Einzelheiten sind im Online‐Supplement, Abbildungen S1 und S2, erläutert.

Die beiden Datensätze wurden in einen Trainingssatz (80%) für die Modellentwicklung und einen Testsatz (20%) für die Validierung aufgeteilt, um sicherzustellen, dass beide Teilsätze die gleiche Klassenverteilung wie die Originaldatensätze beibehielten. Die wesentliche Einschränkung des Datensatzes ist die relative kleine Anzahl an Bildern, wobei einige Klassen unterrepräsentiert sind. Im ersten Modell zeigt sich ein Ungleichgewicht der Klassen bei FD‐ und AGA‐Clustern, während im zweiten Modell die Klasse der AA mit Nachwachsen der Haare im Vergleich zu den anderen Klassen überrepräsentiert ist. Um ausgewogene Datensätze zu erhalten und die Gesamtzahl der Bilder zu erhöhen, wurde eine Datenaugmentation auf die Trainings‐ und Testdatensätze angewendet. Dieser Ansatz umfasst verschiedene Transformationen existierender Daten, einschließlich zufälliger Bildrotation und Farbverfälschung mittels Hauptkomponentenanalyse (*Principal Component Analysis*, PCA).^45^, ^46^, ^47^ Einzelheiten sind im Online‐Supplement erläutert.

### Modellbeschreibung

In unserer Forschung verwendeten wir drei Architekturen von *Convolutional Neural Network* (CNN) Modellen: *Residual Neural Network* (ResNet152), *Dense Convolutional Network* (DenseNet169) und EfficientNetB0.^48^, ^49^, ^50^, ^51^, ^52^ Modelle und Parameterdetails sind im Online‐Supplement und in Tabelle S2 des Online‐Supplements beschrieben.

### Transfer Learning

Aufgrund der eingeschränkten Größe des verfügbaren Bild‐Datensatzes ist es eine Herausforderung ein Modell von Grund auf zu trainieren, trotz der deutlichen Steigerung der Trainingsdaten durch Augmentation. Um das Problem der eingeschränkten Daten zu überwinden, verwendeten wir die Theorie des Transfer Learning (TL) mit vortrainierten Gewichten. Die Einzelheiten zu TL sind im Online‐Supplement und in Abbildung S3 erläutert.^52^, ^53^ Die Modelle wurden unter Verwendung von NVIDIA GeForce‐RTX‐3060 6GB GPUs und der PyTorch‐Bibliothek trainiert.

### Leistungskennzahlen

Zur Bewertung der Leistungsfähigkeit jedes Modells wurden Genauigkeit, *Recall*, Präzision und F1‐Scores sowohl insgesamt als auch für jede Klasse berechnet. Die Genauigkeit ist das Verhältnis korrekt vorhergesagter Richtig/Falsch‐Klassifikationen bei allen Ergebnissen und der F1‐Score ist der harmonische Mittelwert, der sowohl Präzision als auch Recall berücksichtigt. Aufgrund des Ungleichgewichts bei der Klassenverteilung, insbesondere in Stufe‐1, wurde ein gewichteter durchschnittlicher F1‐Score berechnet. Außerdem wurden die Grenzwertoptimierungskurve (*Receiver Operating Characteristic*, ROC) und die Präzision‐Recall‐Kurve (PR) für jede Klasse anhand der Scores der Modelle und einem Einer‑vs.‑Alle‐Ansatz dargestellt. Micro und gewichtete Durchschnittskurven stellen die allgemeine Leistungsfähigkeit des Modells dar. Anschließend wurden die Fläche unter der Kurve (AUC) und die Fläche unter der Präzision‐*Recall*‐Kurve (AUPRC) berechnet. Schließlich wurden die Durchschnittswerte der klinischen Eigenschaften der Patienten bestimmt. Die freiwillige Teilnahme wurde sichergestellt und von allen Teilnehmern wurde eine schriftliche Einwilligungserklärung eingeholt. Diese Studie wurde von der Ethikkommission der Universität Triest genehmigt (Genehmigungscode:70268).

## ERGEBNISSE

In unsere Studie wurden 152 Teilnehmer aufgenommen. Ihre Charakteristika sind in Tabelle 4 zusammengefasst. Der Originaldatensatz umfasste 1196 AA‐ (391 aktiv, 237 inaktiv und 568 mit Nachwachsen), 300 FD‐, 606 FFA‐ und LPP‐, 67 AGA‐ und 1405 GF‐Bilder. Der Testdatensatz enthielt 641 Bilder für Stufe‐1 und 240 Bilder für Stufe‐2. Nach dem Augmentationsprozess erhöhte sich die Anzahl der Bilder auf insgesamt 49 219 für Stufe‐1, wobei alle Krankheitspathologien vertreten waren. Dies umfasste 39 368 Bilder für den Trainingssatz und 9851 Bilder für den Testsatz. Für Stufe‐2, die Bewertung der verschiedenen Aktivitätsniveaus der AA, enthielt der Datensatz 23 885 Bilder, davon 19 076 Bilder für den Trainingssatz und 4809 Bilder für den Testsatz. Die Ergebnisse für die trainierten Modelle für Stufe‐1 und Stufe‐2 sind in den Tabellen 5 beziehungsweise 6 zusammengefasst. Die Leistungsmessgrößen wurden anhand des Testdatensatzes unter Ausschluss der augmentierten Bilder berechnet. F1‐Score und Genauigkeit sind dargestellt.

**TABELLE 4:** Patientencharakteristika.

Variable		Ergebnisse
Gesamtzahl der Patienten n (%)		152 (100)
Geschlecht	Weiblich n (%)	105 (69,1)
	Männlich n (%)	47 (30,9)
Erkrankung der Kopfhaut	AA n (%)	58 (38,2)
	AGA n (%)	20 (13,2)
	FD n (%)	17 (11,1)
	LPP n (%)	19 (12,5)
Gesunde Freiwillige	GF n (%)	38 (25,0)

*Abk*.: AA, Alopecia areata; AGA, Alopecia androgenetica; GF, gesunde Freiwillige; FD, Folliculitis decalvans; FFA, frontale fibrosierende Alopezie; LPP, Lichen planopilaris; n, Anzahl

**TABELLE 5:** Scores der Netzwerkmodelle für die diagnostische Klassifizierung der Alopecia areata – Stufe‐1.

Modell 1 Erkrankung	Kennzahlen	DenseNet169	EfficientNetB0	ResNet152
Gesamt	Genauigkeit	87,36	88,92	86,27
Gesamt	F1‐gewichtet	86,82	88,17	85,4
AA	Präzision	89,87	89,88	87,92
	Recall	88,38	92,12	87,55
	F1	89,12	90,98	87,73
FFA‐LPP	Präzision	86,55	90,09	89,38
	Recall	85,12	82,64	83,47
	F1	85,83	86,21	86,32
FD	Präzision	91,66	94,83	91,53
	Recall	91,66	91,67	90
	F1	91,66	93,22	90,76
GF	Präzision	84,23	86,1	82,02
	Recall	91,12	93,66	91,22
	F1	87,59	89,71	86,37
AGA	Präzision	66,67	50	0
	Recall	14,29	7,14	0
	F1	23,53	12,5	0

*Abk*.: AA, Alopecia areata; AGA, Alopecia androgenetica; GF, gesunde Freiwillige; FD, Folliculitis decalvans; FFA, frontale fibrosierende Alopezie; LPP, Lichen planopilaris

**ABBILDUNG 3:**
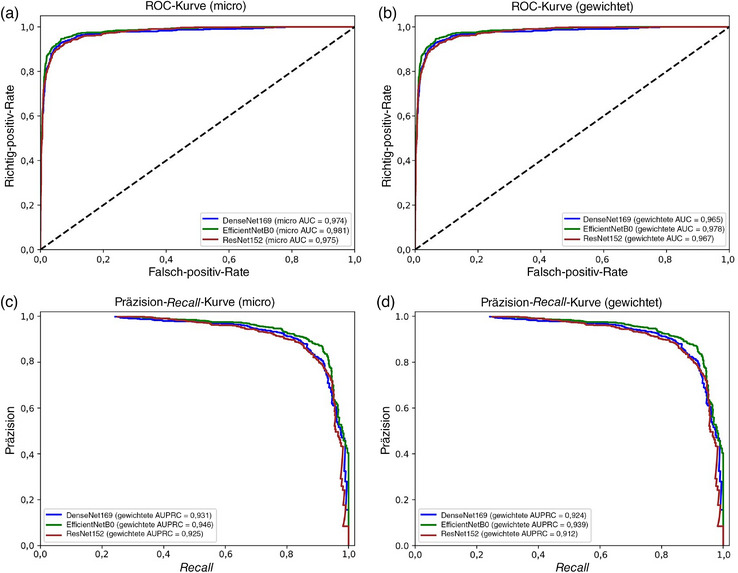
(a) Diagnostische Klassifizierung der Alopecia areata: Stufe‐1 *Receiver Operating Characteristic* (ROC)‐Kurve (micro); (b) Stufe‐1 *Receiver Operating Characteristic* (ROC)‐Kurve (gewichtet); (c) Stufe‐1 Präzision‐*Recall*‐Kurve (micro); (d) Stufe‐1 Präzision‐*Recall*‐Kurve (gewichtet).

**ABBILDUNG 4:**
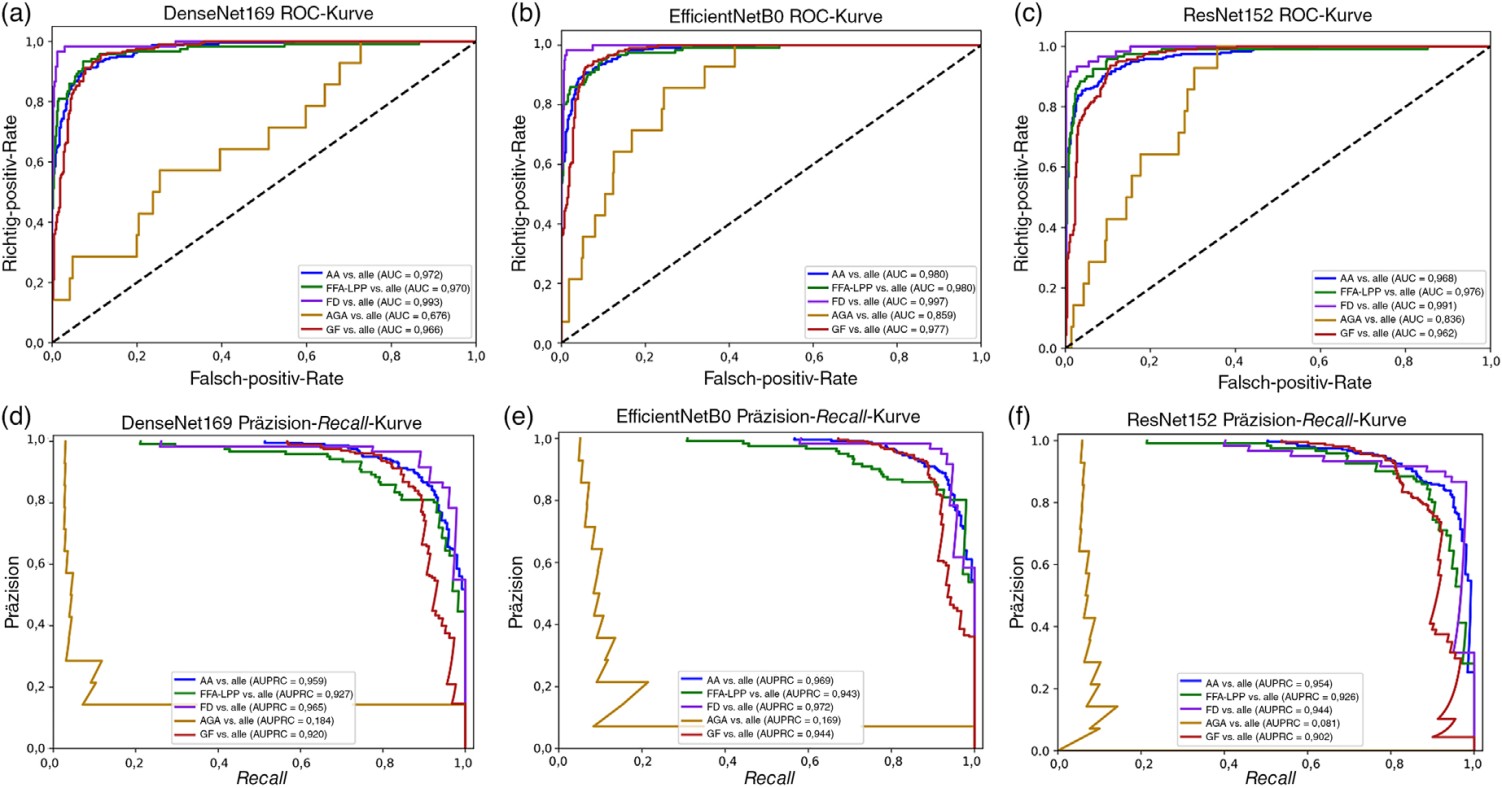
(a) Diagnostische Klassifizierung der Alopecia areata: Stufe‐1 *Receiver Operating Characteristic* (ROC)‐Kurve des DenseNet169‐Modells: jede Klasse vs. alle; (b) Stufe‐1 *Receiver Operating Characteristic* (ROC)‐Kurve von EfficientNetB0: jede Klasse vs. alle; (c) Stufe‐1 *Receiver Operating Characteristic* (ROC)‐Kurve von ResNet152: jede Klasse vs. alle; (d) Präzision‐*Recall* (PR)‐Kurve des DenseNet169‐Modells: jede Klasse vs. alle; (e) Stufe‐1 Präzision‐*Recall* (PR)‐Kurve von EfficientNetB0: jede Klasse vs. alle; (f) Stufe‐1 Präzision‐*Recall* (PR)‐Kurve von ResNet152: jede Klasse vs. alle.

In Stufe‐1 zeigte das Modell EfficientNetB0 die höchsten Gesamtergebnisse, mit einer Gesamtgenauigkeit von 88,92% und einem gewichteten F1‐Score von 88,17%. Die höchste Fähigkeit zur Unterscheidung der AA wurde ebenfalls mit diesem Modell erzielt. Dabei beliefen sich der F1‐Score auf 90,98%, der Recall auf 92,12% und die Präzision auf 89,88%. Vergleichbare Ergebnisse wurden für die Gruppen FD, FFA‐LPP und GF erzielt (Tabelle 5). Alle Modelle erreichten AUCs und AUPRCs > 0,90 (Abbildung 3), wobei die AUCs und AUPRCs für AA vs. alle > 0,95 waren (Abbildung 4). Allerdings wurden signifikante Unterschiede bei der diagnostischen Fähigkeit zwischen den Erkrankungen beobachtet. Dies betraf insbesondere AGA im Vergleich zu den anderen Erkrankungen. Die suboptimale Leistung bei der AGA‐Klasse kann dem sehr geringen Vorliegen im Datensatz zugeschrieben werden (1,8% aller Bilder).

**ABBILDUNG 5:**
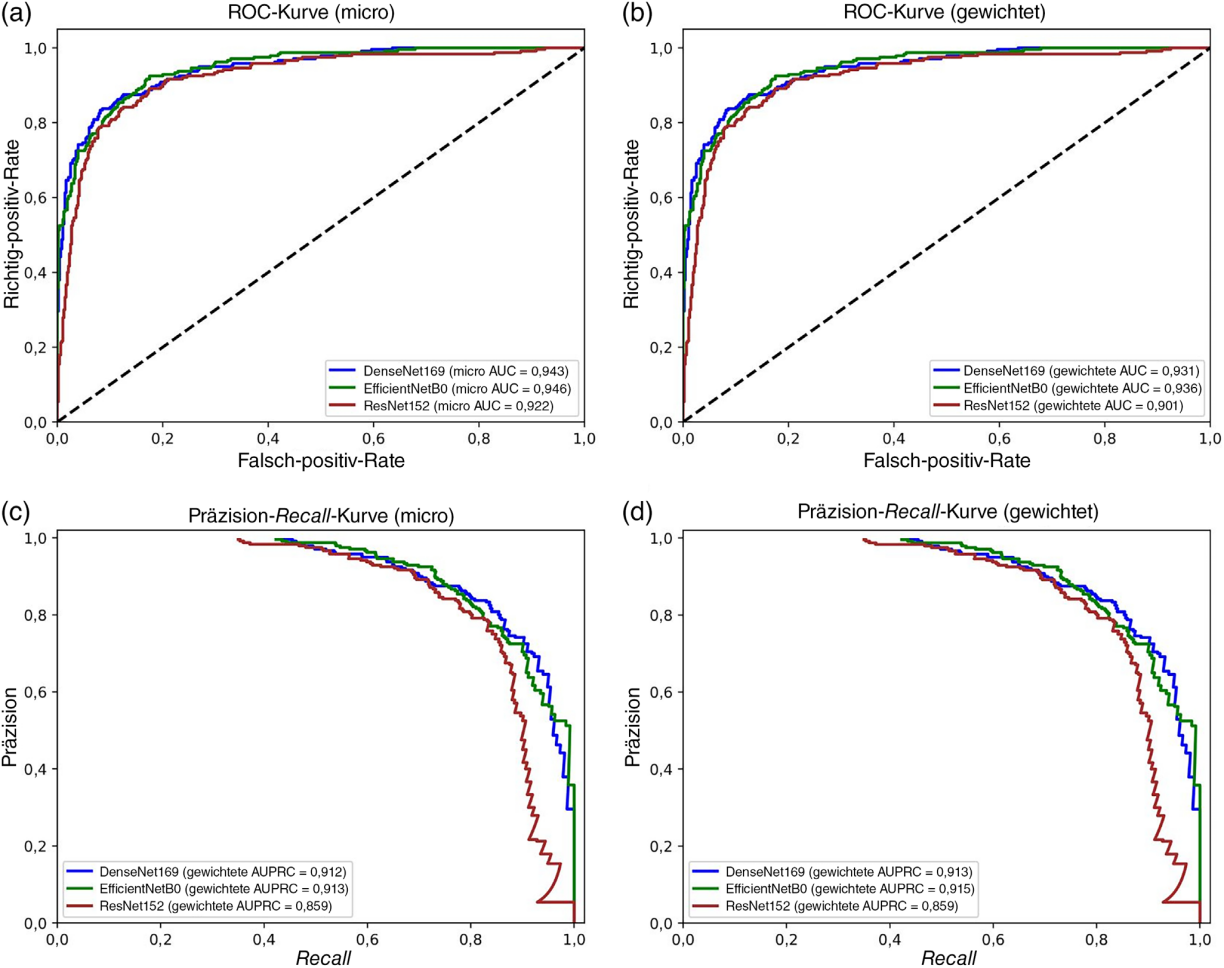
(a) Klassifizierung des Aktivitätsniveaus der Alopecia areata: Stufe‐2 *Receiver Operating Characteristic* (ROC)‐Kurve (micro); (b) Stufe‐2 *Receiver Operating Characteristic* (ROC)‐Kurve (gewichtet); (c) Stufe‐2 Präzision‐*Recall*‐Kurve (micro); (d) Stufe‐2 Präzision‐*Recall*‐Kurve (gewichtet).

**ABBILDUNG 6:**
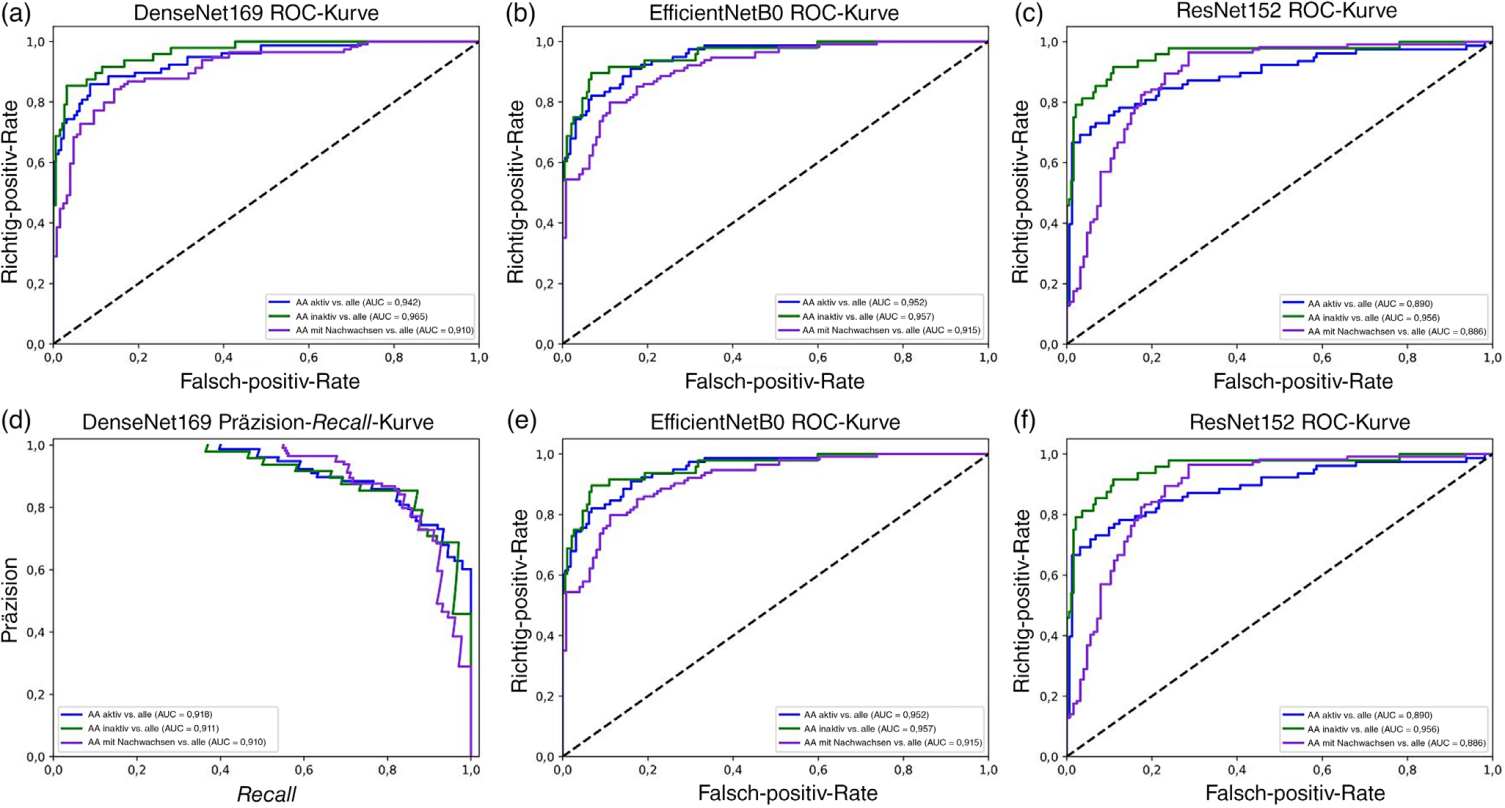
(a) Klassifizierung des Aktivitätsniveaus der Alopecia areata: Stufe‐2 *Receiver Operating Characteristic* (ROC)‐Kurve des DenseNet169‐Modells: jede Klasse vs. alle; (b) Stufe‐2 *Receiver Operating Characteristic* (ROC)‐Kurve von EfficientNetB0: jede Klasse vs. alle; (c) Stufe‐2 *Receiver Operating Characteristic* (ROC)‐Kurve von ResNet152: jede Klasse vs. alle; (d) Präzision‐*Recall* (PR)‐Kurve des DenseNet169‐Modells: jede Klasse vs. alle; (e) Stufe‐2 Präzision‐*Recall* (PR)‐Kurve von EfficientNetB0: jede Klasse vs. alle; (f) Stufe‐2 Präzision‐*Recall* (PR)‐Kurve von ResNet152: jede Klasse vs. alle.

Verglichen mit EfficientNetB0 und ResNet152 wurde in Stufe‐2 die beste Leistung mit dem Modell DenseNet169 beobachtet. Die Gesamtgenauigkeit betrug 83,33% und der gewichtete F1‐Score 83,36%. Das Modell erhielt die ausgeglichene Leistung über alle drei Gruppen aufrecht: aktive AA mit einem F1‐Score von 81,25%, inaktive AA mit einem F1‐Score von 84,44% und AA mit Nachwachsen der Haare mit einem F1‐Score von 84,35% (Tabelle 6). In Stufe‐2 zeigten DenseNet169 und EfficientNetB0 vergleichbare Ergebnisse, mit AUCs und AUPRCs > 0,90. Bei ResNet152 waren AUC und AUPRC > 0,85 (Abbildung 5). Abschließend erzielten die Modelle EfficientNetB0 und DenseNet169 in der Aktivitätsanalyse für jedes Aktivitätsstadium eine höhere AUC und AUPRC als ResNet152 (Abbildung 6).

**TABELLE 6:** Scores der Netzwerkmodelle für die Klassifizierung des Aktivitätsniveaus der Alopecia areata – Stufe‐2.

Modell 2 Aktivität	Kennzahlen	DenseNet169	EfficientNetB0	ResNet152
Gesamt	Genauigkeit	83,33	80,83	79,16
Gesamt	F1‐gewichtet	83,36	80,75	79,13
AA aktiv	Präzision	79,27	90,47	76,00
	Recall	83,33	73,08	73,08
	F1	81,25	80,85	74,51
AA inaktiv	Präzision	90,48	83,33	84,44
	Recall	79,16	72,92	79,17
	F1	84,44	77,78	81,72
AA mit Nachwachsen der Haare	Präzision	83,72	75,56	79,16
	Recall	85,09	89,47	83,33
	F1	84,35	81,92	81,20

*Abk*.: AA, Alopecia areata

## DISKUSSION

KI umfasst jede Technologie, die es Computern ermöglicht, menschliche Fähigkeiten zur Entscheidungsfindung zu replizieren oder zu übertreffen und dabei komplexe Aufgaben unabhängig von oder mit minimaler menschlicher Beteiligung zu bewältigen.^54^, ^55^, ^56^ Im Allgemeinen beziehen sich die Subsets ML und DL auf die Verbesserung der Leistung eines Computerprogramms durch Erfahrung in Bezug auf spezifische Aufgaben und Leistungskennzahlen.^9^, ^57^, ^58^ Künstliche neuronale Netze (KNN) sind ein spezifischer durch die Struktur und Funktion des menschlichen Gehirns inspirierter Typ eines ML‐Modells.^9^, ^57^, ^58^ Tiefe neuronale Netze (*Deep Neural Networks*, DNN) bestehen in der Regel aus mehreren versteckten Ebenen, die in tief verschachtelten Netzwerkarchitekturen organisiert sind. Aufgrund dieser Eigenschaften sind DNN besonders hilfreich in Domänen mit großen und hochdimensionalen Daten. Der Mangel an großen Datensätzen mit markierten dermatologischen Bildern hat DL‐Anwendungen bisher limitiert.^9^, ^57^, ^58^ Obwohl KI‐basierte Klassifizierungssysteme menschliche Experten nicht ersetzen können, haben sie laut Literaturangaben die Fähigkeit, Experten zu unterstützen und ihre Fähigkeit, genaue Hautdiagnosen zu stellen und Erkrankungen zu behandeln, um mehr als 33% zu verbessern.^59^ Tatsächlich haben DL‐Instrumente das Potential, die Effizienz von Arbeitsabläufen auf mehrere Arten zu verbessern. Sie können KI‐gestützte Diagnosen und Differentialdiagnosen schnell und effizient mit den assoziierten Wahrscheinlichkeiten stellen und so Hausärzte bei der Patientenbeurteilung unterstützen. Außerdem können sie Dermatologen bei zeitaufwendigen oder repetitiven Aufgaben entlasten, zum Beispiel bei umfassenden Bewertungen mittels Dermatoskopie. Durch die Automatisierung dieser Aufgaben können Dermatologen klinische Daten wirksamer integrieren und für medizinische Entscheidungsfindungen höherer Ordnung anwenden, einschließlich der Behandlung von Haarerkrankungen.^57^

Bei Haarerkrankungen steigt die Prävalenz der AA weiterhin jährlich an, wobei die Diagnose im Wesentlichen auf der klinischen Beurteilung mit Unterstützung durch Trichoskopie beruht.^60^, ^61^ Dieses Verfahren kann die AA‐Diagnose verbessern, indem sie die Beurteilung der inflammatorischen Aktivität, die Identifizierung von Indikatoren des Schweregrads, die Bewertung der therapeutischen Wirksamkeit und die Bestimmung von Prognosefaktoren unterstützt.^34^ Allerdings kann die Trichoskopie aufgrund der zahlreichen Erkrankungen, die das Haar und die Kopfhaut beeinflussen können, eine Herausforderung darstellen, da viele dieser Erkrankungen ähnliche trichoskopische Merkmale aufweisen (zum Beispiel gelbe Punkte), was die Diagnose erschwert.^62^ Daher wird die Diagnose nicht anhand eines einzigen trichoskopischen Merkmals sondern anhand einer Konstellation von Eigenschaften gestellt. Dies erschwert die Erkennung und Differenzierung dieser Erkrankungen zusätzlich.^8^ Die Tatsache, dass diese Eigenschaften sich mit der Zeit und in Abhängigkeit der Aktivität und des Stadiums der Erkrankung sowie aufgrund der Reaktion auf eine medizinische Behandlung verändern können, macht die Bewertung von Erkrankungen, die fluktuieren oder fortschreiten, noch komplizierter.^7^, ^34^, ^63^ Darüber hinaus können bei den Patienten überlappende Zustände oder atypische klinische Manifestationen auftreten.^64^, ^65^ Daher erfordert Kompetenz bei der Trichoskopie eine Spezialausbildung, was für viele Ärzte ein Hindernis darstellt.^8^ Demzufolge ist die Entwicklung eines KI‐Modells vielversprechend hinsichtlich der Unterstützung von Dermatologen bei der Diagnose und Stadieneinteilung der AA und als Orientierungshilfe für die medizinische Behandlung. Im Jahr 2021 wurde anhand der Klassifizierung klinischer Bilder ein Framework zur Unterscheidung von gesundem Haar und AA entwickelt. ^66^ KI wurde außerdem hinsichtlich der Bewertung des Schweregrads von AA durch ein DL‐Framework untersucht. Tatsächlich konnten Lee et al. zeigen, dass Dermatologen mit einem Computer‐gestützten Ansatz zur Bestimmung des SALT‐Scores eine signifikant höhere Genauigkeit bei der Stadieneinteilung und eine bessere Interrater‐Reliabilität erzielten.^26^ Dieser Ansatz könnte die Aussagekraft des SALT‐Scores für die Vorhersage eines Nachwachsens der Haare erhöhen und so seine Zuverlässigkeit für verschiedene klinische und Forschungszwecke steigern.^67^ Diese Befunde verweisen auf die Bedeutung solcher Modelle als Orientierungshilfe für die klinische Behandlung bei verschiedenen AA‐Patienten. Daher kann die Entwicklung eines Frameworks unter Verwendung videodermatoskopischer Bilder ein hilfreiches Instrument für die Differenzierung der AA von anderen Erkrankungen und zur Erkennung von Aktivitäts‐ oder Inaktivitätsmarkern darstellen. Ein KI‐Modell könnte die medizinische Behandlung durch Erkennung von Zeichen einer AA‐Remission unterstützen, mithilfe derer Ärzte Behandlungen reduzieren oder absetzen oder einen beobachtenden und abwartenden Ansatz verfolgen können. Umgekehrt könnte die KI bei Vorliegen von Aktivitätsmarkern die Entscheidung zum Aufrechterhalten der Therapie oder zur Eskalation von topischen zu systemischen Behandlungen unterstützen.^68^ Eine weitere potentielle Anwendung von DL‐Tools liegt in der Teledermatologie, die darauf abzielt, Barrieren beim Zugang zur fachärztlichen Versorgung zu überwinden. Mithilfe von KI‐Algorithmen könnten periphere Zentren vom Expertenwissen in spezialisierten Zentren profitieren. Solche Algorithmen könnten auch Selbstüberwachungs‐Apps zur Nachverfolgung von Haarerkrankungen ermöglichen.^59^, ^69^, ^70^, ^71^

Unsere Studie präsentiert einen zweistufigen Algorithmus zur Verbesserung der Diagnose anhand einer Reihe von Verfahren zur Datenaugmentation, die für Bilder der Kopfhaut geeignet sind, und eines TL‐Ansatzes. Ziel der Stufe‐1 war die Entwicklung eines Modells, das Bilder der AA von anderen Erkrankungen unterscheiden kann. Dabei wurde mit EfficientNetB0 eine Gesamtgenauigkeit von 88,92% und ein gewichteter F1‐Score von 88,17% erzielt. Konkret für die AA‐Klasse erzielte EfficientNetB0 den höchsten F1‐Score von 90,98%. Die Leistungsfähigkeit des Modells wurde zudem durch ROC‐ und PR‐Kurven nachgewiesen. In Stufe‐2 zeigte das mit DenseNet169 trainierte Modell den höchsten F1‐Score (83,36%) und die höchste Genauigkeit (83,33%). Die Ergebnisse waren über alle Aktivitätsniveaus der Erkrankung hinweg konsistent. AUC und AUPRC waren bei DenseNet169 und EfficientNetB0 vergleichbar. Die Unterschiede zwischen den diagnostischen Fähigkeiten zwischen AA und AGA im Vergleich zu anderen Erkrankungen konnten der begrenzten Anzahl an AGA‐Patienten und folglich AGA‐Bildern zugeschrieben werden. Zukünftige Arbeiten sollten sich auf die Steigerung der Anzahl von Bildern unterrepräsentierter Erkrankungen, wie AGA in unserer Studie, konzentrieren.

Soweit wir wissen, unterscheidet sich unser Modell von bereits in der Literatur beschriebenen Modellen durch die Verwendung von videodermatoskopischen statt klinischen Bildern, um AA von gesunden Personen und anderen trichologischen Erkrankungen zu unterscheiden und die AA in unterschiedlichen Aktivitätsstadien zu bewerten^10^, ^11^, ^12^, ^13^, ^14^, ^15^, ^16^, ^17^, ^18^, ^19^, ^20^, ^21^, ^22^, ^23^, ^24^, ^25^, ^26^, ^27^, ^28^, ^29^, ^30^, ^31^ Unsere Studie hat folgende Limitationen: *(1)* Unterschiede in der diagnostischen Fähigkeit bei verschiedenen Erkrankungen der Kopfhaut aufgrund eines unausgewogenen Datensatzes (zum Beispiel wenige AGA‐Patienten); *(2)* Lücken im Datensatz, zum Beispiel das Fehlen von Erkrankungen wie Tinea capitis oder anderen vernarbenden Alopezien aufgrund des Alters der Patienten als Einschlusskriterium oder einer begrenzten Fallzahl (zum Beispiel LED); *(3)* begrenzte Generalisierbarkeit über verschiedene Prototypen aufgrund der ethnischen Herkunft unserer Patientenpopulation; und *(4)* Abhängigkeit von High‐End‐Geräten (zum Beispiel Medicam 1000^®^), die nicht in allen Krankenhäusern verfügbar sind, was die breitere telemedizinische Anwendung einschränkt.

Zusammenfassend hat DL ein enormes Potential in der Dermatologie als unterstützendes diagnostisches Instrument für Hauterkrankungen mit vielversprechendem Nutzen bei der Unterstützung der Diagnose und Quantifizierung von Erkrankungen. Dieses Potential wurde bereits durch die Klassifizierung zahlreicher anderer Hautläsionen in einer Genauigkeit, die der von Dermatologen entspricht, nachgewiesen. Soweit wir wissen, unterstreicht unsere Studie erstmals die potentielle Anwendung der KI in der Trichoskopie für die Diagnose und die Bestimmung des Aktivitätsniveaus von AA. Diese Anwendung kann genauere Diagnosen ermöglichen sowie die Patientenversorgung und den Arbeitsablauf der Dermatologen verbessern. Weitere Studien sind erforderlich, um diese Algorithmen weiterzuentwickeln und zu validieren und so die Versorgung und Sicherheit der Patienten zu verbessern, die Produktivität der Dermatologen zu steigern und den Zugang zu einer hochwertigen dermatologischen Versorgung zu erweitern.

## DANKSAGUNG

Open access publishing facilitated by Universita degli Studi di Trieste, as part of the Wiley ‐ CRUI‐CARE agreement.

## INTERESSENKONFLIKT

Alle anderen Autoren erklären, dass kein Interessenkonflikt besteht. R.D.C.C. hat Vortragshonorare von Novartis, Eli Lilly, Sanofi und Pfizer erhalten. I.Z. ist Mitglied eines Daten‐ und Sicherheitsüberwachungsgremiums für Philogen und hat Vergütungen oder Honorare für Vorträge, Präsentationen, das Verfassen von Manuskripten oder Weiterbildungsveranstaltungen von Sanofi Genzyme, Sunpharma, Novartis, MSD, BMS, Philogen, Biogena, La Roche Posay, Kyowara Kirin, Fotofinder, Mallinckrodt, Cieffe Derma, Pierre Fabre, Regeneron, Canova, Almirall und Beiersdorf sowie Unterstützung für die Teilnahme an Konferenzen und/oder Reisen von Difa Cooper erhalten.

## Supporting information



Supplementary information

Supplementary information

Supplementary information

Supplementary information
